# Burning Mouth Syndrome Treated with Low-Level Laser and Clonazepam: A Randomized, Single-Blind Clinical Trial

**DOI:** 10.3390/biomedicines12051048

**Published:** 2024-05-09

**Authors:** Ana Garcia Martinez, Pia Lopez-Jornet, Luis Pardo Marin, Eduardo Pons-Fuster, Asta Tvarijonaviciute

**Affiliations:** 1 Department of Dermatology, Stomatology, Radiology and Physical Medicine, Faculty of Medicine, University of Murcia, Hospital Morales Meseguer Clinica Odontologica Marques Velez S/N, 30008 Murcia, Spain; ana.garcia27@um.es; 2 Interdisciplinary Laboratory of Clinical Analysis, Interlab-UMU, Regional Campus of International Excellence ‘Campus Mare Nostrum’, University of Murcia, Espinardo, 30100 Murcia, Spain; lpm1@um.es (L.P.M.); asta@um.es (A.T.); 3 Department Anatomy, Faculty of Medicine, University of Murcia, El Palmar, 30120 Murcia, Spain; eduardo.p.f@um.es

**Keywords:** burning mouth syndrome, saliva, interleukins, low-level light therapy, clonazepam

## Abstract

Objective: Burning mouth syndrome (BMS) is a chronic pain disorder characterized by intraoral burning or dysaesthetic sensation, with the absence of any identifiable lesions. Numerous treatments for BMS have been investigated, though without conclusive results. An analysis was conducted of the efficacy of treatment with a low-level diode laser and clonazepam in patients with BMS, and a study was carried out on the levels of different salivary biomarkers before and after treatment. Material and methods: A randomized, single-blind clinical trial was carried out involving 89 patients divided into the following groups: group 1 (laser, The Helbo^®^ Theralite Laser 3D Pocket Probe + clonazepam) (n = 20), group 2 (sham laser placebo) (n = 19), group 3 (laser) (n = 21) and group 4 (clonazepam) (n = 18). Symptom intensity was scored based on a visual analogue scale (VAS). Sialometry was performed before and after treatment, and the Xerostomia Inventory, Oral Health Impact Profile-14 (OHIP-14) and Mini-Nutritional Assessment (MNA) questionnaires were administered. The following markers were measured in saliva samples: interleukins (IL2, IL4, IL5, IL6, IL7, IL8, IL1β, IL10, IL12, IL13, IL17, IL21 and IL23), proteins (MIP-3α, MIP-1α and MIP-1β), GM-CSF, interferon gamma (IFNγ), interferon-inducible T-cell alpha chemoattractant (ITAC), fractalkine and tumor necrosis factor α (TNFα). Results: A significant decrease in the VAS scores was observed after treatment in group 1 (laser + clonazepam) (*p* = 0.029) and group 3 (laser) (*p* = 0.005). In turn, group 3 (laser) showed a decrease in the salivary concentration of fractalkine (*p* = 0.025); interleukins IL12 (*p* = 0.048), IL17 (*p* = 0.020), IL21 (*p* = 0.008), IL7 (*p* = 0.001) and IL8 (*p* = 0.007); proteins MIP1α (*p* = 0.048) and MIP1β (*p* = 0.047); and TNFα (*p* = 0.047) versus baseline. Following treatment, group 1 (laser + clonazepam) showed significant differences in IL21 (*p* = 0.045) and IL7 (*p* = 0.009) versus baseline, while group 4 (clonazepam) showed significant differences in IL13 (*p* = 0.036), IL2 (*p* = 0.020) and IL4 (*p* = 0.001). No significant differences were recorded in group 2 (sham laser placebo). Conclusions: The low-level diode laser is a good treatment option in BMS, resulting in a decrease in patient symptoms and in salivary biomarkers. However, standardization of the intervention protocols and laser intensity parameters is needed in order to draw more firm conclusions.

## 1. Introduction

Burning mouth syndrome (BMS) is a chronic disorder defined by the International Headache Society (IHS) as an intraoral burning or dysaesthetic sensation without visible lesions capable of accounting for the patient discomfort. The symptoms manifest during at least two hours a day and persist over time for over three months with no apparent lesions [1]. The burning sensation is usually found in the anterior two-thirds or tip of the tongue [2]. The chronic pain, together with the lack of efficacy of the prescribed treatments, has an impact upon the mood state of the patient, giving rise to or worsening psychiatric disorders such as anxiety or depression and adversely affecting quality of life [3].

The prevalence of BMS in the general population varies between 0.1 and 3.9%, depending on the diagnostic criteria used. The syndrome is more common in women than in men, generally manifesting in menopause or post-menopause, between 50 and 70 years of age [4].

The exclusion of other local or systemic conditions that could account for intraoral burning is a very important component of the diagnosis of BMS, which remains a diagnosis of exclusion and often a diagnostic challenge [2].

The underlying etiopathogenesis is not clear, though a multifactorial etiology has been suggested, involving interactions between local, systemic and psychological factors [5]. On one hand, neurophysiological studies suggest that BMS is neuropathogenic, involving dysfunction of the peripheral and central nervous pathways [6,7]. Another etiological hypothesis refers to hormonal status, since BMS is more frequent in menopausal or post-menopausal women [8]. In turn, mention must be made of the psychological factors that modulate patient perception of the burning, itching and pain symptoms. In this regard, studies have evidenced an association between anxiety and depression in patients with BMS, since psychological alterations have been seen to exacerbate the symptoms in the context of chronic pain [9].

The management of BMS remains a challenge for clinicians. A range of therapies have been evaluated, with controversial results, and no clear conclusions have been drawn as to what constitutes effective treatment. What does seem clear is that a multifactorial therapeutic approach is needed, with the aim of adequately addressing all the factors involved. A number of pharmacological and non-pharmacological strategies have been found to improve patient pain and quality of life. In this context, drugs such as anxiolytics or antidepressants have been shown to be effective in managing the symptoms of BMS, with clonazepam being the most widely used drug [10].

In relation to the non-pharmacological strategies, minimally invasive treatments have been investigated in recent years, including low-level diode laser (light) therapy (LLLT). This technique is well accepted by both patients and clinicians, as it is a painless procedure, and many studies have reported improvements in patient quality of life following its use as an alternative for producing BMS symptom relief. The fundamental principle underlying LLLT is the acceleration of tissue regeneration and wound healing, with a decrease in inflammation and pain [11,12,13,14]. The use of LLLT reduces the concentration of different biomarkers, such as nuclear factor kappa B (NF-kB), tumor necrosis factor alpha (TNFα), cyclooxygenase-2 (COX-2) and interleukin IL1β [15].

Saliva is a body fluid that reflects the physiological condition of the body. The sampling of saliva is easily achieved, noninvasive and proves relatively inexpensive; it therefore constitutes a good indicator for obtaining further data on BMS [5]. Many salivary markers can be used to assess physiological responses. The most widely analyzed are cytokines, which are proteins and glycoproteins produced by different types of cells and that fundamentally act as regulators of immune responses. Other markers are growth factors corresponding to different types of cells, particularly hematopoietic cells. Studies of saliva in patients with BMS have reported increases in different biomarkers, such as interleukins (IL2, IL6, IL1β) and TNFα [16,17]. Cytokines are associated with nociceptive signaling and are usually increased in nociplastic pain disorders including BMS [18,19]. The meta-analysis by Porporatti et al. suggests more stress factors in questionnaire-based studies, and higher levels of cortisol, α-amylase, IgA and IL8 biomarkers were found in patients with BMS than in the control group [20].

Considering the above, the working hypothesis of the present study was that the use of LLLT and clonazepam is able to reduce both the pain symptoms and burning sensation in patients with BMS and the concentration of different salivary biomarkers. Specifically, an analysis was conducted on the efficacy of treatment with LLLT and clonazepam in patients with BMS, along with an evaluation of the biomarkers present in the saliva of these individuals.

## 2. Material and Methods

### 2.1. Study Design and Patient Selection

**Figure 1:**
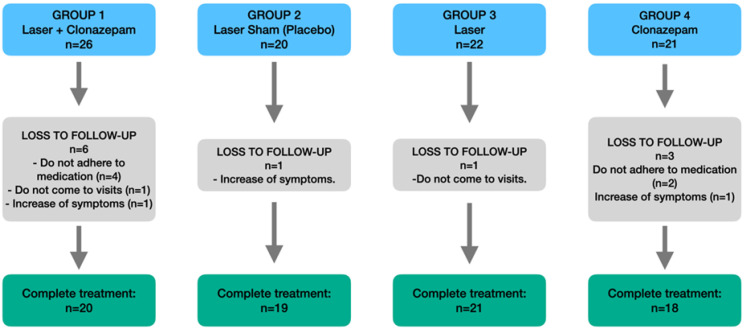
Study flow chart.

A randomized, single-blind clinical trial was carried out in 89 patients (Figure 1), of which 78 completed the treatment in Hospital Clínico Universitario Morales Meseguer (Murcia, Spain). The study was carried out following the Consort Statement (http://www.consort-statement.org/). All the participants were informed about the study and gave consent to participation following approval of the trial by the local Ethics and Biosafety Committee (ID: 407/2021). The clinical registry of this study was also obtained (NCT06217731).

The following inclusion criteria were established: patients over 18 years of age diagnosed with BMS according to the definition of the IHS [1], with continuous burning and itching symptoms in the absence of any justifying cause for a minimum of 6 months, with bilateral presentation in the oral cavity and no clinical alterations of the oral mucosa. Patients who did not suffer from anemia or deficiencies of vitamin B12 or folic acid were included in the study. Patients without periodontal disease and patients who had not received any treatment for BMS symptoms in the previous 3 months were also included. Pregnant or nursing patients were excluded, as were oncological patients, individuals requiring changes in their systemic medications and patients with oral disorders other than BMS, such as Sjögren’s syndrome, candidiasis, lichen planus or ongoing infections.

The patients were divided into groups as follows: group 1 (n = 20) (patients subjected to low-level diode laser treatment [Helbo^®^ Theralite Laser 3D Pocket Probe] once a week for one month and with clonazepam 0.25 mg once every 24 h for one month); group 2 (n = 19) (patients treated with the same laser once a week for one month but without activating the laser tip, as a placebo); group 3 (n = 21) (patients treated only with the laser once a week for one month); and group 4 (n = 18) (patients treated only with clonazepam 0.25 mg once every 24 h for one month). The patients were unaware of the different groups on which the study was based.

### 2.2. Data Collection

The participants were evaluated by a single qualified professional (AGM) who compiled a case history of each patient, with sociodemographic and clinical data (diseases, medications, body mass index [BMI]) and information on habits (alcohol, smoking). An extraoral and intraoral examination was carried out to assess the points where the patients experienced burning and itching sensation. The intensity of these symptoms was scored using a visual analogue scale (VAS) from 0 (absence of the symptom) to 10 (maximum symptom intensity possible), in order to assess increases or decreases in the intensity of the symptoms. All patients underwent sialometry to assess salivation. The saliva samples were collected for 5 min under resting conditions, and salivation was classified as normal (≥0.4 mL) or pathological (≤0.4 mL). The patients in turn completed the Xerostomia Inventory, in which 11 questions explore dry mouth sensation, with higher scores corresponding to greater dry mouth sensation (0–55 points) [21]. Perceived oral health was assessed using the Oral Health Impact Profile-14 (OHIP-14), based on a series of questions that yielded a net total score of 0–56 points, where higher scores corresponded to poorer perceived oral health [22]. Lastly, nutritional habits and BMI were explored with the Mini-Nutritional Assessment (MNA), where a score of 24–30 points indicated normal nutritional status, 17–23.5 points risk of malnutrition and <17 points corresponded to malnutrition [23]. These tests were performed in each group before treatment and after four weeks of treatment to compare variations in the intensity of symptoms, salivation, perceived oral health and nutritional habits.

### 2.3. Treatments Applied

The present randomized, controlled clinical trial involved four treatment groups. Patient assignment to each group was performed on a blind basis using a random sequence-generating program (https://www.randomizer.org). The sealed and numbered envelopes containing the group assignments were prepared by a team member not involved in patient inscription or treatment. The envelopes were opened sequentially only after each patient was registered in the study.

- Low-level light therapy (LLLT):

The Helbo^®^ Theralite Laser 3D Pocket Probe (Bredent Medical GmbH & Co. KG, Senden, Germany) was applied once a week for one month. Following the instructions of the manufacturer, we treated each zone of the oral cavity that presented symptoms using the Helbo^®^2D Spot Probe (Bredent Medical GmbH & Co. KG, Senden, Germany), allowing it to act for 30 s per light spot (active surface area 19 mm^2^; energy density = 30 s × 200 mW/cm^2^ = 6 J/cm^2^). The location that was most frequently reported in the patient was the tongue.

- Sham laser (placebo):

The Helbo^®^ Theralite Laser 3D Pocket Probe was applied but without activating the Helbo^®^2D Spot Probe, as the sham treatment of each zone of the oral cavity that presented symptoms, for 30 s per light spot.

- Clonazepam:

Rivotril (clonazepam) 0.25 mg was prescribed as follows: the patients were instructed to disintegrate the tablet in the mouth at bedtime, after oral hygiene, moving the fragments over the zones that presented symptoms. Despite this rinse, some small tablet fragments were not completely dissolved and therefore remained in the saliva. This residue was subsequently expelled by the participants.

### 2.4. Saliva Collection

The saliva samples were collected using a standardized technique. The patients remained under resting conditions for one hour before sampling. The unstimulated saliva was collected using the drainage method for 5 min; ensuring that all saliva produced was systematically collected for analysis. The samples were obtained in approximately the same time window (9:00–12:00 a.m.) and were centrifuged (3000 rpm for 10 min at 4 °C) immediately after collection. The supernatant was transferred to polyethylene tubes and stored at −80 °C until analysis [24].

### 2.5. Biochemical Testing of the Saliva Samples

Salivary inflammatory markers were measured by analyzing cytokine levels with the Milliplex^®^ Map Human High Sensitivity T Cell Panel Premixed 21 system following the manufacturer’s instructions [25].

### 2.6. Statistical Analysis

Basic descriptive statistics were recorded in the form of frequencies and the mean (m), median (Me), standard deviation (sd) and range (R). The normality of the data was assessed with the Shapiro–Wilk test.

The use of nonparametric tests was decided after assessing homoscedasticity of variance and homogeneity of the sample distribution. The chi-square test was applied to explore differences in the sociodemographic variables and to compare other variables of clinical interest such as alcohol consumption and smoking. No attempt was made to transform the data using the logarithm before deciding to use a nonparametric test. The distribution abnormality did not seem to respond to a negatively skewed distribution, so it was not considered the best option. The Mann–Whitney U-test in turn was used to explore pre–post differences within groups, while the Kruskal–Wallis test was used to assess pre–post differences between groups. The threshold for statistical significance was considered as *p* < 0.05.

Previous studies in patients with BMS have been able to detect statistically significant differences with ≤20 patients per group. Considering these data, we assumed that our study had sufficient statistical power to meet its objectives (groups 1, 2, 3 and 4 with n = 20, n = 19, n = 22 and n = 18, respectively) [11,26,27,28]. Sample size calculation was carried out using G*Power (version 3.1.9.4; Heinrich-Heine-Universität Düsseldorf, Düsseldorf, Germany). The minimum number of participants for significant comparisons between the four groups was 76 subjects, assuming an alpha error of 0.05.

## 3. Results

In Figure 1, the flowchart of the study is shown. The descriptive analysis (Table 1) showed the study groups to be homogeneous, with no significant differences in terms of age or gender distribution, alcohol consumption, smoking or BMI. However, the differences among the groups in terms of the duration (evolution) of BMS were statistically significant (*p* = 0.022). Specifically, a duration of the syndrome of 1–5 years was seen to predominate, while evolutive periods of over 10 years were practically not observed among the groups.

The comparison of the pre–post treatment parameters of the different questionnaires (Table 2) showed a significant decrease in the VAS scores after treatment in group 1 (laser and clonazepam) (*p* = 0.029) and group 3 (laser) (*p* = 0.005). The parameters corresponding to the rest of the questionnaires showed no significant differences.

Table 3 presents the results for the salivary biomarkers. Cytokine GM-CSF showed post differences (*p* = 0.010) between groups, with lower mean levels in group 1 (laser and clonazepam), followed by group 2 (sham laser), group 3 (laser) and finally group 4 (clonazepam).

Group 3 (laser) showed significant salivary reductions in fractalkine (*p* = 0.025); interleukins IL10 (*p* = 0.057), IL12 (*p* = 0.048), IL17 (*p* = 0.020), IL1β (*p* = 0.054), IL21 (*p* = 0.008), IL7 (*p* = 0.001) and IL8 (*p* = 0.007); MIP1α (*p* = 0.048) and MIP1β (*p* = 0.047); and TNFα (*p* = 0.047).

Group 1 (laser and clonazepam 0.25 mg) only showed significant differences in IL21 (*p* = 0.045) and IL7 (*p* = 0.009) after treatment. In turn, group 4 (clonazepam) showed significant differences in interleukins IL13 (*p* = 0.036), IL2 (*p* = 0.020) and IL4 (*p* = 0.001), with a decrease in mean values after treatment. Lastly, group 2 (sham laser) showed no significant differences in any of the salivary markers after treatment.

## 4. Discussion

At present, the management strategies for burning mouth syndrome (BMS) seek to reduce pain and improve quality of life and reducing patient anxiety or stress. Nevertheless, none of the existing treatment options are truly effective. The present study was therefore carried out to evaluate the efficacy of low-level diode laser (light) therapy (LLLT) as an alternative for the management of BMS along with clonazepam, assessing the levels of different cytokines in saliva samples collected before and after treatment. We found that treatment with LLLT resulted in a decrease in 8 of the 21 biomarkers evaluated in our study (fractalkine, IL12, IL17, IL21, IL7, MIP1α, MIP1β and TNFα). In comparison, treatment with LLLT and clonazepam resulted in a decrease in only two biomarkers (IL21 and IL7), while treatment with clonazepam alone resulted in an increase in the concentration of IL13, IL2 and IL4.

In relation to the underlying mechanisms of action, clonazepam is a benzodiazepine and a γ-aminobutyric acid (GABA) receptor agonist. This receptor is widely distributed in the central nervous system and in peripheral tissues. The action of clonazepam upon these receptors may have beneficial effects for patients with BMS. Pure, small-fiber peripheral neuropathy may be better controlled with local clonazepam, while the central mechanisms may benefit more from systemic clonazepam [29]. Benzodiazepines have quite a few and significant side effect profiles. One of the debilitating side effects is their addictive potential along with dizziness, drowsiness, prolonged reaction time, impaired muscle coordination, tiredness or fatigue, among others [30]. On the other hand, LLLT is a rapidly growing technology that is being used to treat a range of disorders that require tissue stimulation for regeneration, the reduction in pain and inflammation and the restoration of function. The mucosa responds well to the operating wavelength of LLLT, as the photons are absorbed by the mitochondrial chromophores in the mucosal cells, incrementing electron transport, the release of nitric oxide and adenosine triphosphate, blood flow and the generation of reactive oxygen species. The cells are thus activated, which results in improved tissue repair and healing. The noninvasive nature of this technique and the almost total lack of side effects encourage its use in treatments of this kind. In the case of LLLT, no side effects are described [31].

The present study showed that LLLT using the Helbo^®^ Theralite Laser 3D Pocket Probe at a setting of 6 J/cm^2^ = 200 mW/cm^2^ for 30 s at each point of the oral mucosa presenting symptoms, applied once a week for one month in patients with BMS, resulted in a significant decrease in pain intensity (*p* = 0.005) in group 3 (laser). The VAS scores evidenced better outcomes than in group 1 (laser with clonazepam 0.25 mg), where significant differences were also recorded (*p* = 0.029), though with a less pronounced decrease in symptom intensity. In both group 1 and group 3, the decrease in symptoms after treatment was greater than in group 2 (placebo) and group 4 (clonazepam).

Visual analogue scales are the main tools used by studies designed to measure pain symptoms in patients with BMS. Arbabi-Kalati used low-level laser irradiation at 630 nm/30 mW for 10 s per target zone twice a week for four weeks in patients with BMS. The patients were divided into two groups (including a placebo group), and significant improvements were recorded in the VAS scores corresponding to burning sensation. Likewise, significant differences in the OHIP-14 scores were observed after laser application [13]. Similar results were obtained by Bardellini et al. using the K Laser Cube 3 once a week for 10 weeks at 660–970 nm/6.4 mW in 90 patients randomized to the placebo or active laser treatment. Significant reductions were observed in the VAS scores corresponding to pain and burning sensation, associated with a significant decrease in the OHIP-14 scores, with improved patient quality of life [32]. In contrast, Sikora et al., using a Gallium Aluminum Arsenide laser at 12 J/cm^2^ = 830 nm/100 mW for 5 min in the form of 10 sessions over two weeks, documented significant improvement of burning sensation as evidenced by the VAS scores, but no significant differences in patient quality of life were observed according to the OHIP-14 [33]. On the other hand, Suguya et al. and Spanemberg et al. recorded no significant differences in the VAS scores after treatment on comparing the active laser and the sham laser groups, thus evidencing the great placebo effect which LLLT may have [26,34].

The study of cytokine levels in saliva thus may reveal substantial information on the ongoing processes. Studies comparing salivary biomarkers in patients with BMS versus healthy controls have reported significant increases in IL1β among the individuals with BMS versus the controls [35]. A recent systematic review including 8 quality studies on salivary markers in healthy individuals and in patients with BMS has concluded that the salivary IL6 levels are higher among the patients with BMS, though other studies have reported no significant differences in terms of this parameter. Similar considerations apply to IL2, while in the case of IL10 the selected studies failed to record significant differences [18].

In the present study, upon analyzing the biomarkers in saliva, we recorded a decrease in the levels of IL21 and IL7 after treatment in group 1 (laser with clonazepam). These are proinflammatory cytokines, and a lowering of their concentration after treatment thus evidences a decrease in inflammation. In group 3 (laser), treatment resulted in a decrease in fractalkine and in interleukins IL12, IL17, IL21, IL7 and IL8; in proteins MIP1α and MIP1β; and in TNFα. In contrast, the concentrations of IL13, IL2 and IL4 increased in group 4 (clonazepam). In this regard, IL13 and IL4 are anti-inflammatory cytokines; both induce the differentiation of B lymphocytes and act jointly with the macrophages, reducing the effects of the proinflammatory cytokines IL1, IL6 and IL8. Consequently, the anti-inflammatory component is favored, though contrarily IL2 is a proinflammatory cytokine, and an increase in the concentration of this molecule thus worsens inflammation. These results are in accordance with previously reported studies in which patients with BMS treated with LLLT (5 sessions a week for one month, in the form of 7-min applications at 1 J/cm^2^ = 30 mW) reported significant reductions in salivary IL6 and TNFα (*p* > 0.001) [36]. Furthermore, Lu et al. conducted a meta-analysis comparing different lasers with operating wavelengths between 600 and 1100 nm. Longer wavelengths of 780–950 nm have greater penetration and were used for deep tissues, while wavelengths of 600–700 nm were used for superficial tissues. Both approaches were seen to be of similar effectiveness in reducing pain and burning sensation. These wavelengths favor improvement of cytokine levels, promoting nerve recovery and leading to treatment success, with a decrease in cytokine expression (IL1β, IL6, IL8 and TNFα) and the induction of a beneficial biomodulation effect [37]. Overall, these data suggest that cytokines play a very important role in pain, acting through different mechanisms at various sites in the pain transmission pathways [38].

Significant reductions in the largest number of salivary biomarkers appeared to correspond to group 3 (laser), followed by group 1 (laser with clonazepam); however, all the markers where laser therapy combined with clonazepam produced positive results also responded favorably to laser treatment alone. The administration of clonazepam therefore does not seem to be important in relation to any of the analyzed markers, with the exception of IL2, IL13 and IL4, where the post-treatment levels were seen to increase. In group 2 (placebo), no differences were recorded after treatment for any of the salivary biomarkers.

Based on the above data, clonazepam as sole treatment can increase inflammation, and when the drug is used in combination with laser treatment, the biomarker-reducing effect of the latter decreases, since laser therapy as a sole treatment shows superior results in terms of the reduction in biomarkers to the combination of laser therapy with clonazepam. These data could be conditional on patient adherence to treatment, since clonazepam use was solely dependent upon the patient, while laser therapy implied patient agreement with the clinician in the correct application of the therapy in the clinic. Nevertheless, further large-scale studies should be performed to confirm these observations.

In general, the treatment of BMS is focused in the same way as that of other neuropathic pain conditions. Clonazepam is used to reduce the symptoms of burning mouth syndrome. In a study of 60 patients with BMS divided into two groups (clonazepam 0.5 mg dissolved under the tongue versus a group using a tongue protector [placebo]), significant differences were observed after treatment, with the clonazepam group showing improvement after one month as assessed by the VAS scores [29].

In the systematic review carried out by Tan et al., studies using topical clonazepam reported a decrease in burning sensation, though other studies failed to record significant differences versus the control group [4].

One of the effects of LLLT is the improvement of peripheral circulation and blood oxygenation and the elimination of toxic products. Scardina et al. applied LLLT at a setting of 800 nm/60 mW for 8 sessions twice a week for four consecutive weeks. Forty patients with BMS were randomly assigned to LLLT or the placebo. The capillaries were evaluated using capillaroscopy, with the observation of significant differences after treatment; a reduction in capillary size was associated with improvement of the symptoms, while the placebo group showed no differences between baseline and post-treatment [39].

The meta-analysis carried out by Camolesi et al. involving studies in patients with BMS subjected to LLLT showed improvements in both pain (VAS score) and in patient quality of life (OHIP-14 score) [12].

However, it should be mentioned that the studies found in the literature show important heterogeneity in terms of the protocols used, with differences in the LLLT operating parameters and in the duration and form of application of the treatments. This makes it particularly difficult to establish comparisons between the different studies.

The main limitation of this study is the complex and multifactorial etiopathogenesis of BMS, which requires the conduction of more in-depth research. The therapeutic outcomes of LLLT appear to be slightly more consistent than those afforded by clonazepam, with the absence of side effects, though solid data remain lacking. Further clinical studies comparing LLLT and placebos are needed to more clearly define the role of the former in the management of BMS, with the standardization of protocols in order to obtain more conclusive data. On the other hand, in view of the scientific evidence of the relationship between BMS and the levels of different cytokines, the latter could be used as biomarkers of the disease with which to evaluate treatment effectiveness [16]. Another limitation of the study was time; the follow-up period was short, and the study should continue in the long term. The authors intend to conduct the study for a longer period, and we recognize that the number of patients included in this study could be considered low, which could affect the generalizability of the findings. Further studies with larger sample sizes are recommended to validate these results.

## 5. Conclusions

The results of the present study suggest that LLLT is a good treatment option, reducing pain and burning sensation in patients with BMS and lowering the levels of salivary inflammatory markers. Further large-scale studies would be needed to confirm these findings.

## Figures and Tables

**Table 1:** Description of the study sample.

Variable	Group 1 Laser + Clonazepam	Group 2 Sham Laser (Placebo)	Group 3 Laser	Group 4 Clonazepam	*p*
**Gender** n (%)	0.569 ^c^				
Females	18 (90)	19 (100)	20 (95.2)	17 (94.4)	
Males	2 (10)	0 (0)	1 (4.8)	1 (5.6)	
**Age** m (sd)	61.55 (11.62)	62.11 (10.91)	64.43 (11.67)	63.06 (11.23)	0.842 ^a^
**Smoking** n (%)	0.730 ^c^				
1–10 cigarettes	2 (10)	3 (15.8)	2 (9.5)	2 (11.1)	
11–20 cigarettes	1 (5)	2 (10.5)	0 (0)	1 (5.6)	
+20 cigarettes	1 (5)	0 (0)	0 (0)	0	
Ex-smoker	5 (25)	3 (15.8)	7 (33.3)	2 (11.1)	
Non-smoker	11 (55)	11 (57.9)	12 (57.1)	13 (72.2)	
**Alcohol** n (%)	0.785 ^c^				
Once a week	1 (5)	1 (5.3)	2 (9.5)	0 (0)	
Weekends	2 (10)	2 (10.5)	1 (4.8)	1 (5.6)	
Daily	1 (5)	0 (0)	0 (0)	0 (0)	
None	16 (80)	16 (84.2)	18 (85.7)	17 (94.4)	
**BMI** m (sd)	25.74 (3.56)	25.67 (4.35)	26.53 (5.95)	25.78 (4.21)	0.975 ^b^
**Evolution** n (%)	**0.022** ^c^				
1–5 years	11 (55)	16 (84.3)	13 (61.9)	14 (77.8)	
6–10 years	7 (25)	3 (15.9)	6 (28.6)	2 (11.2)	
>10 years	4 (20)		2 (9.6)	2 (11.2)	
**Locations** n (%)	0.859 ^c^				
1–3 locations	13 (65)	11 (57.9)	15 (71.4)	11 (61.1)	
4–6 locations	5 (25)	7 (36.9)	5 (23.8)	6 (33.3)	
7–10 locations	2 (10)	1 (5.3)	1 (4.8)	1 (5.6)	

^a^, Student’s *t*-test; ^b^, Mann–Whitney U-test; ^c^, Pearson’s chi-square test; figures in boldface indicate significant differences. n (%), sample size and percentage. m (sd), mean and standard deviation. BMI, body mass index. *p*, probability value.

**Table 2:** Evaluation of differences (pre–post) after four weeks of treatment.

		Group 1 Laser + Clonazepam	Group 2 Sham Laser (Placebo)	Group 3 Laser	Group 4 Clonazepam	*p*
**VAS**	Pre m (sd)	7.20 (2.39)	7.52 (1.92)	7.28 (2.70)	7.05 (2.15)	0.922
	Post m (sd)	5.10 (3.37)	6.52 (2.31)	4.80 (2.73)	5.94 (2.43)	0.236
	*p*	**0.029**	0.157	**0.005**	0.156	
**Xeros**	Pre m (sd)	30.20 (12.72)	25.84 (10.39)	25.14 (14.10)	24.88 (10.71)	0.506
	Post m (sd)	28.35 (13.33)	24.94 (11.63)	23.47 (13.51)	22.61 (10.45)	0.481
	*p*	0.656	0.804	0.698	0.523	
**OHIP-14**	Pre m (sd)	19.95 (8.02)	22.00 (9.03)	17.19 (8.02)	23.94 (9.05)	0.102
	Post m (sd)	18.65 (9.47)	22.15 (10.84)	16.38 (8.61)	20.88 (859)	0.313
	*p*	0.642	0.961	0.754	0.307	
**MNA**	Pre m (sd)	25.07 (3.55)	23.60 (3.89)	24.57 (3.75)	24.63 (3.49)	0.590
	Post m (sd)	25.10 (3.56)	23.65 (3.90)	24.57 (3.75)	24.55 (3.52)	0.633
	*p*	0.947	0.954	1.00	0.888	

Pre, results before treatment. Post, results after four weeks of treatment. m (sd), mean and standard deviation. *p*, probability value. Kruskal–Wallis for between-group differences and the Mann–Whitney U-test for within-group differences. Figures in boldface indicate significant differences. VAS, visual analogue scale. Xeros, Xerostomia Inventory. OHIP-14, Oral Health Impact Profile-14. MNA, Mini-Nutritional Assessment.

**Table 3:** Analysis of salivary biomarkers.

		Group 1 Laser + Clonazepam	Group 2 Sham Laser (Placebo)	Group 3 Laser	Group 4 Clonazepam	*p*
**ITAC**	Pre Me (R)	31.30 (124.24 ± 3.40)	31.03(310.96 ± 19.03)	35.75(192.96 ± 7.52)	35.24(208.95 ± 8.52)	0.893
	Post Me (R)	29.74 (93.03 ± 3.40)	39.59(13.04 ± 1.08)	18.55(187.79 ± 5.57)	36.29(276.95 ± 10.74)	0.066
	*p*	0.134	0.281	0.309	0.571	
**GM_CSF**	Pre Me (R)	1.65(5.27 ± 0.86)	1.97(13.04 ± 1.08)	2.35(13.37 ± 1.20)	2.13(6.80 ± 0.86)	0.456
	Post Me (R)	1.35(4.45 ± 0.65)	1.47(7.10 ± 0.99)	1.54(13.04 ± 0.97)	3.27(6.80 ± 0.99)	**0.010**
	*p*	0.265	0.337	0.131	0.091	
**Fractalkine**	Pre Me (R)	394.89(3537 ± 38.74)	367.79(1379 ± 93.96)	442.14(4895 ± 55.91)	500.13(1827 ± 44.44)	0.901
	Post Me (R)	139.36(1278 ± 38.74)	283.50(1040 ± 86.70)	226.9(974.81 ± 67.45)	222.72(1471 ± 53.27)	0.338
	*p*	0.060	0.118	**0.025**	0.331	
**IFN**γ	Pre Me (R)	7.38(34.11 ± 4.63)	10.54(315.90 ± 3.59)	11.62(105.80 ± 4.63)	9.10(26.45 ± 5.78)	0.264
	Post Me (R)	7.38(32.54 ± 2.44)	6.38(28.84 ± 2.44)	10.65(161.41 ± 2.44)	9.21(19.96 ± 2.44)	0.106
	*p*	0.951	0.244	0.630	0.774	
**IL10**	Pre Me (R)	6.24(25.01 ± 3.09)	8.90(126.96 ± 3.94)	8.90(265.17 ± 3.70)	6.74(20.95 ± 3.09)	0.170
	Post Me (R)	4.86(19.06 ± 4.86)	6.74(44.17 ± 2.84)	5.9(36.14 ± 3.09)	8.79(18.39 ± 3.93)	0.093
	*p*	0.190	0.557	0.057	0.544	
**MIP3**α	Pre Me (R)	21.56(93.92 ± 1.25)	32.85(432.47 ± 14.42)	40.35(216.23 ± 8.73)	32.88(61.56 ± 3.55)	0.248
	Post Me (R)	15.39(103.53 ± 1.68)	32.85(65.94 ± 9.34)	11.87(62.91 ± 3.26)	42.86(184.79 ± 2.44)	**0.008**
	*p*	0.541	0.277	**0.022**	0.070	
**IL12**	Pre Me (R)	0.98(2.97 ± 0.65)	1.08(2.32 ± 0.65)	1.24(3.95 ± 0.65)	1.10(2.72 ± 0.65)	0.335
	Post Me (R)	0.98(1.78 ± 0.52)	0.98(1.95 ± 0.65)	1.13(1.78 ± 0.71)	1.13(2.32 ± 0.94)	0.024
	*p*	0.117	0.185	0.048	0.709	
**IL13**	Pre Me (R)	1.05(7.17 ± 0.49)	1.31(119.70 ± 0.61)	1.98(229.25 ± 0.76)	1.33(6.86 ± 0.48)	0.176
	Post Me (R)	0.76(3.10 ± 0.36)	1.11(96.39 ± 0.33)	0.93(29.28 ± 0.33)	3.34(5.29 ± 0.33)	**0.001**
	*p*	0.081	0.887	0.338	0.036	
**IL17**	Pre Me (R)	2.41(13.35 ± 1.49)	2.75(31.06 ± 1.77)	3.07(16.58 ± 1.77)	2.57(7.25 ± 1.77)	**0.129**
	Post Me (R)	1.91(10 ± 1.49)	2.57(8.02 ± 1.48)	2.57(5.64 ± 1.61)	2.56(5.93 ± 1.61)	0.031
	*p*	0.249	0.223	**0.020**	0.592	
**IL1**β	Pre Me (R)	42.35(2090 ± 0.42)	119.70(2780 ± 2.41)	192.78(2290 ± 3.95)	103.46(662.62 ± 10.80)	0.387
	Post Me (R)	18.89(1189 ± 0.18)	63.18(1610 ± 2.32)	75.95(557.19 ± 0.62)	136.15(1874 ± 2.61)	0.053
	*p*	0.360	0.479	0.054	0.178	
**IL2**	Pre Me (R)	0.22(1.16 ± 0.08)	0.32(0.99 ± 0.07)	3.80(0.98 ± 0.10)	0.34(0.75 ± 0.03)	0.242
	Post Me (R)	0.21(0.47 ± 0.08)	0.28(0.98 ± 0.05)	0.28(0.59 ± 0.08)	0.51(1.03 ± 0.04)	**0.001**
	*p*	0.148	0.865	0.097	**0.020**	
**IL21**	Pre Me (R)	2.54(9.50 ± 0.38)	2.83(6.99 ± 0.52)	2.98(14.62 ± 0.70)	2.26(6.03 ± 0.70)	0.222
	Post Me (R)	1.37(4.65 ± 0.27)	1.99(5.22 ± 0.38)	1.99(4.35 ± 0.27)	2.46(6.03 ± 0.38)	0.316
	*p*	**0.045**	0.222	**0.008**	0.921	
**IL4**	Pre Me (R)	5.26(23.20 ± 3.54)	6.60(2955 ± 4.17)	8.15(2045 ± 2.45)	6.44(12.20 ± 3.54)	0.521
	Post Me (R)	5.26(49.2 ± 2.45)	6.60(788.34 ± 3.54)	5.26(234.38 ± 3.54)	14.40(32.60 ± 5.26)	0.001
	*p*	0.763	0.492	0.381	**0.001**	
**IL23**	Pre Me (R)	37.78(301.9 ± 16.06)	44.19(2634 ± 16.06)	52.88(7370 ± 16.06)	37.78(191.59 ± 16.06)	0.303
	Post Me (R)	27.23(336.4 ± 16.06)	37.78(169.52 ± 16.06)	37.78(749.95 ± 19.51)	50.75(200.07 ± 27.23)	**0.002**
	*p*	0.337	0.303	0.329	0.195	
**IL5**	Pre Me (R)	1.04(1.76 ± 0.87)	1.04(24.10 ± 0.79)	1.35(31.59 ± 0.87)	1.04(2.17 ± 0.64)	0.115
	Post Me (R)	0.88(1.57 ± 0.72)	1.04(8.90 ± 0.75)	1.18(6.05 ± 0.72)	1.04(1.7 ± 0.96)	0.067
	*p*	0.063	0.507	0.307	0.807	
**IL6**	Pre Me (R)	2.44(17.1 ± 0.29)	3.64(218.20 ± 0.79)	2.80(102.24 ± 0.61)	4.10(20.14 ± 0.79)	0.617
	Post Me (R)	1.54(5.49 ± 0.16)	4.32(54.55 ± 0.62)	2.44(35.37 ± 0.42)	4.24(14.87 ± 0.58)	**0.007**
	*p*	0.064	0.370	0.156	0.727	
**IL7**	Pre Me (R)	7.61(34.6 ± 0.62)	7.94(38.57 ± 3.28)	8.84(30.72 ± 1.20)	7.28(19.71 ± 1.46)	0.527
	Post Me (R)	4.43(11.2 ± 0.62)	8.52(21.93 ± 2.82)	4.88(11.22 ± 0.83)	8.52(94.77 ± 1.46)	**0.001**
	*p*	**0.009**	0.379	**0.001**	0.126	
**IL8**	Pre Me (R)	1416(7373 ± 0.60)	2025(6364 ± 376.21)	2350(9923 ± 156.25)	1610(5921 ± 471.61)	0.372
	Post Me (R)	676.10(3440 ± 0.51)	1464(5474 ± 370.55)	1203(2861 ± 159.67)	2320(8962 ± 235.81)	**0.015**
	*p*	0.104	0.314	**0.007**	0.320	
**MIP1**α	Pre Me (R)	8.03(102.42 ± 0.23)	5.21(17.18 ± 0.23)	9.79(114.13 ± 0.68)	5.49(25.41 ± 0.17)	0.106
	Post Me (R)	2.27(26.08 ± 0.20)	3.14(22.46 ± 0.20)	5.67(16.76 ± 0.17)	7.66(29.68 ± 0.27)	0.553
	*p*	0.310	0.787	**0.048**	0.828	
**MIP1**β	Pre Me (R)	2.86(50.35 ± 1.47)	2.82(62.53 ± 1.47)	3.85(57.34 ± 1.11)	2.99(24.63 ± 0.89)	0.893
	Post Me (R)	1.87(26 ± 0.60)	2.99(26.29 ± 2.48)	2.13(23.85 ± 1.07)	2.71(20.27 ± 1.55)	0.082
	*p*	0.302	0.458	**0.047**	0.687	
**TNF**α	Pre Me (R)	10.69(187.98 ± 0.31)	19.34(447.08 ± 2.48)	33.96(254.57 ± 1.79)	18.4(96.05 ± 3.82)	0.104
	Post Me (R)	6.71(392.59 ± 0.24)	22.02(138.80 ± 1.73)	15.24(168.68 ± 1.49)	17.07(116.72 ± 1.01)	**0.005**
	*p*	0.976	0.508	**0.047**	0.785	

Pre, results before treatment. Post, results after four weeks of treatment. Me (R), Median and range. *p*, probability value. Figures in boldface indicate significant differences. Kruskal–Wallis for between-group differences and the Mann–Whitney U-test for within-group differences. ITAC, interferon-inducible T-cell alpha chemoattractant. GM-CSF, granulocyte multifactorial colony stimulating factor. IFNγ, interferon gamma. IL, interleukin. MIP1α, macrophage inflammatory protein alpha. MIP1β, macrophage inflammatory protein beta. TNFα, tumor necrosis factor alpha.

## Data Availability

The datasets generated and/or analyzed during the current study are not publicly available due contains sensitive data that can identify but are available from the corresponding author on reasonable request.
